# Evaluation of a Pilot mHealth Intervention to Engage Primary Care Clients at an Urban Clinic Serving Marginalized Populations: Mixed-Methods Cohort Study

**DOI:** 10.2196/68561

**Published:** 2025-11-27

**Authors:** Lauren Harrison, Christina Fulton, Antonio Marante Changir, Adedayo Ajidahun, Cassidy Tam, Wendy Zhang, Tian Shen, Rolando Barrios, Julio Montaner, Kate Salters, Richard T Lester, Surita Parashar, David M Moore

**Affiliations:** 1 British Columbia Centre for Excellence in HIV/AIDS, #608-1081 Burrard Street, Vancouver, BC, V6T 1Y6, Canada, 1 604-806-3478; 2 Facutly of Medicine, University of British Columbia, Vancouver, BC, Canada

**Keywords:** text messaging, primary health care, marginalized populations, substance use, mental health, mHealth, mobile phone

## Abstract

**Background:**

Many individuals in urban low-income settings face barriers to engaging in primary care and experience systemic challenges such as homelessness and discrimination in the health care system. This study was conducted in the Downtown Eastside of Vancouver, Canada, a low-income neighborhood with intersecting structural vulnerabilities and disproportionate rates of substance use disorders. Advancements in mobile health expand options for facilitating communication between primary care providers and clients.

**Objective:**

We conducted a pilot project that provided primary care clients a mobile phone and access to WelTel, a mobile health tool that uses a 2-way texting approach and sends weekly automated check-in messages. Our study measured phone retention, defined as retaining a study-supplied device and being reachable on the study-supplied device over a 6-month period and explored the acceptability and feasibility of WelTel among a cohort of clients with complex health challenges.

**Methods:**

Stratified random sampling was used to recruit participants from a larger cohort study of primary care clinic clients in the Downtown Eastside. The sample was stratified to ensure equal participation based on gender and Indigenous and non-Indigenous participants. In this mixed methods research, participants completed 3 surveys over a 6-month period from November 2022 to May 2023. The surveys assessed phone retention and functionality as well as phone use, including use of the WelTel platform. Clients who had access to a functional mobile phone after the follow-up period were invited to complete an in-person interview. The semistructured interviews explored clients’ experiences with WelTel, primary care, and engagement with technology.

**Results:**

We enrolled 49 participants (median age 48 y; 53% women and 49% Indigenous) and interviewed 16 participants. A total of 44 clients completed the 6-month survey, and of those clients, 26 (59%) had a functional phone. However, only 14 (29%) clients retained the mobile device supplied during the study and completed the 6-month survey. Phone retention or access to a nonstudy phone was lower among those who had used opioids (18% for both) in the past 3 months (*P*<.01), as well as those who reported not having access to a cellphone at enrollment (25% and 15%, respectively) compared to those who did have phone access at enrollment (*P*=.05). Both the surveys and semistructured interviews indicated that WelTel was generally well received; 25 (96%) of those with a functional phone reported that they liked receiving the weekly messages. During qualitative interviews, the WelTel intervention was reported to strengthen client-provider relationships and create pathways for receiving care.

**Conclusions:**

Overall, the pilot study found this intervention feasible and acceptable to clients; however, barriers to phone retention were an ongoing challenge. Expanded enrollment in the WelTel service will allow us to examine whether it also facilitates engagement in primary care among marginalized urban populations.

## Introduction

Client engagement in primary health care can improve health outcomes and lower health care costs [1]. All Canadian residents receive health care through Canada’s publicly funded health care system, which covers physician and hospital services [2]. However, disparities in access to regular primary care have been growing for marginalized groups in Canada [3]. Primary care visits and continuity of care have decreased from 1999 to 2018, whereas emergency room visits have increased, and this dichotomy has been the strongest in lower-income neighborhoods [4]. People who use drugs (PWUDs) have been found to have lower primary health care engagement, despite complex chronic health needs and high acute emergent care access [5,6]. Better engagement in primary health care with a regular physician among PWUD has been shown to reduce the use of emergency services [7]. Strong provider-client relationships established by building rapport and trust can facilitate patient engagement and retention for those facing systemic barriers to health care engagement [8,9].

Virtual health care, including telehealth and mobile health (mHealth), expanded during the SARS-CoV-2 pandemic, during which Canadian provinces and territories placed lockdowns on residents to mitigate infectious disease spread in 2020 [10]. The lockdowns necessitated virtual care options as movement was restricted, and therefore funding was increased for these modalities; while virtual care peaked during these lockdowns, it appears to have decreased in 2021 [10]. Before the SARS-CoV-2 pandemic, mHealth interventions were already being recognized as innovative tools to support disease self-management, health behavior change, and communication with health care providers [11]. One-way messaging symptoms, where participants receive but do not respond to messages, can nudge users toward behaviors such as prescription refills, and the use of chatbots is being explored as artificial intelligence in health care expands [12].

WelTel is an mHealth tool that provides a secure virtual health platform and allows for 2-way texting between health care providers and clients [13]. The platform was found to place a low burden on health care practitioners with a modest relative cost [14]. WelTel has been used for HIV care in Canada and East Africa and has been found to improve medication adherence, connectedness to care, and health outcomes for people living with HIV [15-17]. In addition to HIV care, previous pilot projects evaluating WelTel in British Columbia and Alberta, Canada, have shown promising results; WelTel was well received by health care providers and patients at a tuberculosis clinic [18], improved asthma control outcomes for patients with physician-diagnosed asthma [19], and was found to support high-quality care among rheumatoid arthritis patients [20]. Among Indigenous youth in British Columbia who use substances, there was interest in using mHealth; however, mobile phone ownership was recognized as a potential barrier to the use of WelTel for clients facing multiple structural vulnerabilities [21].

The Downtown Eastside (DTES) is a neighborhood in Vancouver, Canada, that is disproportionately impacted by poverty, unstable and inadequate housing, and food insecurity [22]. As of 2016, the life expectancy in the DTES was 62.2 years for females and 60.2 years for males compared to 86.6 and 82.5 years, respectively, within the general population of Metro Vancouver [23]. Additionally, the median income in 2016 was CAD $23,352 (US $17,963) in the DTES, whereas the median income in the City of Vancouver was $65,423 [24]. High rates of conditions such as HIV, hepatitis C virus, and mental health and substance use disorders are found among many intersecting social inequities [22]. While a strong sense of community can be found among residents in the DTES, stigma and discrimination are common and can act as a barrier to accessing primary health care [25,26]. Indigenous peoples face health care discrimination and racism and are more likely to reside in the DTES, with approximately 10% of DTES residents identifying as Indigenous compared to 2% in the City of Vancouver [27]. In the DTES, PWUDs have reported multiple barriers to accessing primary health care [5]. As such, this population may benefit greatly from enhanced interventions to improve access to primary health care.

Previous qualitative research from our team indicated that mHealth would be desirable in this community and may help to mitigate barriers to accessing primary care [26]. We conducted a pilot project that supplied clients of one primary care clinic in the DTES with a mobile phone and data, talk, and text mobile phone plan as well as access to the WelTel service over a 6-month period. We used quantitative methods to measure phone retention, defined as retaining the study-supplied device and being reachable on the study-supplied device, phone usage, and client experiences of the WelTel platform. Qualitative methods were used to garner a deeper understanding of user experiences, tech engagement, and health care experiences. This mixed methods approach allowed us to generate insight into the feasibility and acceptability of the program.

## Methods

### Setting

In October 2019, the Hope to Health Clinic was opened to provide interdisciplinary primary health care to residents of the DTES with unmet health needs who were previously unattached to primary care [28]. The Hope to Health Clinic was designed to engage and retain clients facing multiple barriers to accessing primary health care and to improve health outcomes for this population [28]. Clients at the Hope to Health Clinic receive low-barrier psychosocial and primary care services to address health, mental health, and addiction challenges [29]. The innovative work at the Hope to Health Clinic is connected to onsite research teams that allow for collaboration between clinicians, researchers, and community members.

### Study Sample

Beginning June 2021, all clients of the Hope to Health Clinic were offered enrollment in the Hope to Health Engagement and Retention Evaluation (HERE) study, a longitudinal cohort study consisting of a baseline survey and follow-up surveys every 6 months for 3 years. A convenience sample of clients who attended the clinic and were interested in the study was enrolled; posters at the Hope to Health Clinic informed clients of their eligibility for the study, medical practitioners facilitated connections between clinic clients and the research team, and research team members were available for face-to-face conversations. At enrollment, participants in the HERE study were given the option to consent to being contacted by the research team for the purpose of participating in a substudy on mobile phones and the WelTel intervention.

Of those who provided this consent, a stratified random sample of participants was selected using random allocation in Microsoft Excel. Participants were stratified based on self-identified Indigenous or non-Indigenous status and self-identified gender to ensure equal opportunities for participation across these strata. All transgender participants in the HERE study were offered enrollment in the WelTel pilot. Additionally, all participants who identified as nonbinary or other gender identities were offered enrollment, but none enrolled. Study staff made outreach attempts to selected participants to offer enrollment, and the random selection procedure was repeated until enrollment was completed with a target of 50 participants. All participants were supplied with a mobile phone device and registered in the WelTel platform. The mobile phones had touchscreens and additional software functions, including internet and the option to download numerous games and applications. All phones were previously used with Android operating systems and had been rebuilt and certified by the provider. A member of the research team provided instructions on how to use the mobile phone and WelTel; ensured the participant had password protected their device; and answered any questions that arose about the phone, the data plan, or WelTel. During this session, participants also consented to complete interviewer-administered surveys every 2 months for a 6-month follow-up period (3 surveys total). Clients who no longer had the study-supplied device but provided their own mobile phone during the follow-up period were able to register their new device with the phone plan and WelTel. Participants were enrolled from November 2022 to May 2023 with follow-up continuing until November 2023.

### Intervention

Participants received a weekly automated check-in message from WelTel that asked “how are you?” On the basis of participants’ responses, WelTel categorized the messages as “okay,” “not okay,” or “unrecognized.” Messages labeled “unrecognized” were not automatically designated by the WelTel system as okay or not okay. Participants were instructed to minimize personal information shared with WelTel when responding to the initial check-in messages. Medical office assistants monitored incoming messages and responded according to the need expressed in the participant’s message, including messages that were unrecognized by the system. Participants who did not reply to the original check-in message did not receive further follow-up from clinic staff for that week.

This study used a mixed methods design to evaluate provisioning of the phone and the acceptability of the WelTel messaging system. Quantitative surveys were used to characterize phone retention and functionality, general phone use, WelTel use, and clinic communication. Qualitative interviews elucidated a deeper understanding of WelTel accessibility and user experiences as well as tech engagement and primary care experiences.

### Data Collection and Analysis

#### Surveys

Following enrollment in the pilot project, participants completed bimonthly surveys for 6 months with a Peer Research Associate (PRA). PRAs are trained researchers with lived experience of the marginalization experienced by residents of the DTES. Surveys were completed over the phone or in person at the Hope to Health Clinic. The surveys took approximately 10 minutes.

HERE study baseline surveys were used to describe participant characteristics and phone access and to assess interest in using technology to engage with health care. Phone retention was summarized using the pilot project follow-up survey data, and *χ*^2^ and Fisher exact tests were used to assess HERE study baseline variables associated with phone retention. Descriptive statistics from the follow-up surveys and the WelTel platform show WelTel usage and client satisfaction with WelTel.

#### Semistructured Interviews

At the end of the 6-month follow-up period, all participants who had a functional mobile phone were offered enrollment in the qualitative substudy up to a maximum of 20 participants. Interviews were completed at the Hope to Health Clinic and were facilitated in pairs by a PRA and the Research Coordinator. Participants provided consent and completed the semistructured interview in 1 session, which lasted approximately 30 to 45 minutes. All interviews were audio recorded and transcribed verbatim. After completing each interview, the Research Coordinator and PRA debriefed about the interview and discussed early observations of emergent codes.

We used Braun and Clarke’s [30] thematic analysis to provide a flexible framework for identifying themes and took a codebook approach [31]. Two research coordinators trained in qualitative analysis began by open-coding 3 transcripts and identifying emergent codes. PRAs provided insight and feedback as these emergent codes were organized into the codebook. Using the codebook, all interviews were coded independently in NVivo (Lumivero, Denver, CO) by both research coordinators, and the research coordinator who co-facilitated all interviews acted as the primary coder. The primary and secondary coders frequently collaborated to refine the codebook and found a high degree of consensus between their codes.

### Ethical Considerations

Ethics approval was obtained from the University of British Columbia/Providence Health Care Research Ethics Board (#H20-03256). All clients provided informed consent for the quantitative surveys at enrollment in the study and had the option of indicating their interest in the qualitative interviews. Clients who agreed to take part in the qualitative interviews provided informed consent through a separate process before the interview took place. Clients were made aware that they could withdraw from the study at any time. The data were deidentified and securely stored. Participants were provided a CAD $15 ($US 10.80) honorarium per survey and a CAD $30 ($US 21.60) honorarium for the qualitative interview. While financial incentives may influence participants’ willingness to participate in research, we felt that it was appropriate to reimburse participants for their time, as is standard research practice. Additionally, the participants were provided mobile phones to participate in this study, which were considered their personal property and not expected to be returned. The talk/text/data plans were provided to participants for up to 2 years, provided they retained their phone provided by the study.

## Results

### Quantitative Surveys

#### Baseline Characteristics

Of the 49 participants who enrolled in the pilot study, 26 (53%) identified as women and 23 (47%) identified as men inclusive of cisgender and transgender participants. The median age was 48 years (IQR 37-56). Nearly half (n=24, 49%) of participants identified as Indigenous, 20 (41%) identified as White, and 5 (10%) identified as other ethnicities. At the time of the HERE study baseline survey, 44 (90%) participants reported an annual income below $20,000, and 12 (25%) participants were currently homeless; an additional 9 (18%) participants had been homeless in the previous 3 months. A total of 19 (39%) participants were taking medications to treat opioid use disorder (ie, methadone), 24 (49%) participants used opioids, 21 (43%) participants used stimulants, and 16 (33%) participants reported injecting substances in the previous 3 months.

The HERE study baseline data indicated 27 (55%) of the participants enrolled in the phone study had regular access to a cell phone, and 11 (22%) had used a cell phone to text their health care provider before enrollment in the pilot. Thirty-eight (78%) participants indicated they would like to text with their health care provider; of these participants, 33 (87%) were interested in receiving appointment reminders, 31 (82%) in receiving health information, and 31 (82%) in texting to set up appointments. Additionally, 38 (78%) participants indicated they would like to be able to use a mobile phone to remind the clinic about their prescription refills, and 35 (71%) participants reported they would like to use a mobile phone to have a clinic visit using video.

### Follow-Up Surveys and WelTel Data

Of the 49 participants enrolled in the pilot study, 46 (94%) completed at least one of 3 follow-up surveys, and 44 (90%) completed the third follow-up survey at the end of the 6-month period. Of these 44 participants, 14 (32%) had retained their study-supplied mobile phone, and an additional 12 (27%) participants had access to another functional phone (Table 1). Of the 30 participants who did not retain their phone, 28 (64%) reported that their study supplied mobile phone had been stolen. No participants reported that their phone had been sold or damaged. While we found similar proportions of participants with no access to a phone at the end of the study based on age (44% of participants aged ≥50 y vs 39% for those aged <50 y), a higher proportion of those aged ≥50 years had retained their phone (44% vs 25%), but a lower proportion had access to a nonstudy phone at the end of the pilot (13% vs 36%; *P*=.04 for all comparisons). Phone retention or access to a nonstudy phone was lower among those who had used opioids (18% for both) in the past 3 months compared to those who did not (45% and 36% respectively; *P*<.01). As well, those who reported not having access to a cellphone at the HERE study baseline survey also had lower proportions of phone retention or access to a nonstudy phone (25% and 15%, respectively) compared to those who did have phone access at enrollment (38% for both; *P*=.05). Stimulant or alcohol use in the past 3 months, homelessness, ethnicity, and gender were not associated with phone retention and access to a functional phone (Table 1).

**Table 1.** Factors associated with phone retention and access after 6 months of follow-up.

Variable	Total	Retained the study supplied device (n=14), n (%)	Access to a functional phone (not the study supplied device; n=12), n (%)	No access to a functional phone (n=18), n (%)	*P* value
Age (y)					
19‐49	28	7 (25)	10 (36)	11 (39)	
50‐65+	16	7 (44)	2 (13)	7 (44)	.04
Gender					
Male	20	7 (35)	4 (20)	9 (45)	
Female	24	7 (29)	8 (33)	9 (38)	.64
Ethnicity					
Indigenous	21	9 (43)	3 (14)	9 (43)	
Caucasian/White	18	4 (22)	6 (33)	8 (44)	
Other ethnicity	5	1(20)	3 (60)	1 (20)	.22
Homelessness					
Currently homeless	11	4 (36)	0 (0)	7 (64)	
Homeless, past 3 mo (not current)	9	2 (22)	3 (33)	4 (44)	
Homeless, more than 3 mo ago	17	5 (29)	8 (47)	4 (24)	
Never been homeless	7	3 (43)	1 (14)	3 (43)	.13
Regular access to a cell phone at baseline					
No	20	5 (25)	3 (15)	12 (60)	
Yes	24	9 (38)	9 (38)	6 (25)	.05
Opioid use in the past 3 mo					
No	22	10 (45)	8 (36)	4 (18)	
Yes	22	4 (18)	4 (18)	14 (64)	.01
Stimulant use in the past 3 mo					
No	24	10 (42)	7 (29)	7 (29)	
Yes	20	4 (20)	5 (25)	11 (55)	.18
Alcohol use in the past 3 mo					
No	28	11 (25)	6 (21)	11 (39)	
Yes	16	3 (19)	6 (38)	7 (18)	0.31

At the third bimonthly survey, 25 (96%) of the 26 participants with a functional phone reported that they liked receiving the weekly WelTel text messages. Across the 3 follow-up surveys, participants reported 16 unique incidents of responding to a WelTel message that they needed help; 7 (44%) of these incidents were pertaining to a medical problem, and 7 (44%) participants needed help with appointment reminders. Of these incidents, participants reported 12 (75%) of their problems were fully addressed. WelTel data showed that 643 (37%) of responses from clients indicated that they were okay, 75 (4%) indicated that the client was not okay or the response type was unrecognized, and 1027 (59%) of check-in messages received no response (Table 2). The median response time for clients to reply to the WelTel message was 221 minutes (Q1-Q3: 23‐1415 min).

**Table 2.** Frequency and type of WelTel response. The median of the total number of texts sent by clients over the study period was 30 (Q1 to Q3: 0-45 ).

Type of response	Vaues, n (%)	
Not okay	17 (1)	
Okay	643 (37)	
Unrecognized (not designated as not okay or okay)	58 (3)	
No response	1027 (59)	

### Semistructured Interviews

A total of 16 participants who had access to a functional phone at the third bimonthly survey completed the qualitative interviews. Of the 16 participants who completed in-depth interviews, 7 (44%) were men, and 9 (56%) were women; 6 (38%) identified their ethnicity as Caucasian/White; 8 (50%) identified as Indigenous; and 2 (13%) identified as other ethnicities. The median age was 48.5 years (Q1-Q3: 47-56 y). At the time of participant enrollment in the WelTel pilot study, 4 (29%) were currently homeless, 4 (29%) were not homeless but had been homeless in the previous 3 months, 4 (29%) had previously been homeless more than 3 months before, and 2 (14%) had never been homeless. Another 2 had missing information on homeless status.

Many participants who completed a qualitative interview viewed the WelTel platform as a tool to support health. Two key themes emerged: client-provider connection and program improvement. Communication and care, as well as self-assessment, were identified as important aspects of the client-provider connection.

### Client-Provider Connection

#### Communication and Care

The importance of the relationship between clients and their providers was emphasized by participants throughout this study, and feeling cared for was frequently mentioned as a benefit of the WelTel program. While one participant described feeling that:

*someone who cares is on the other side.*

[Man, Indigenous]

Another participant voiced that:

*It makes me feel like they’re concerned and that they care even a little bit even though I think it’s a robot.*

[Woman, Indigenous]

Having weekly texts from the clinic was well received and fostered positive relationships with the clinic staff; the automation of the initial check-in text did not interfere with this relationship building between clients and providers.

Many participants expressed that WelTel facilitated “open and honest” communication with their primary care providers and removed feelings of judgment. The improved communication appeared to both contribute to and result from strengthened client-provider relationships. Increased comfort disclosing medical issues through WelTel provided participants with a pathway to receive care that they may not have otherwise accessed. This was explained by a participant when she compared her health care before using WelTel to her experience with the platform:

*I wouldn’t call. I would just deal with whatever was going on, whether I had a small stroke or whatever. I would just deal with it. Now with the WelTel I feel like I can communicate with them without feeling embarrassed.*

[Woman, Indigenous]

By attenuating negative feelings around accessing medical care, participants were empowered to engage in their healthcare. This process was further described by the participant:

*I have an abscess and it’s got me scared and they sent the [WelTel] text asking me how I was and I told them I was in a lot of pain, but otherwise I was doing good and they texted back. And that led to talking to a nurse and now I’m going to get medical assessment done because of that text.*

[Woman, Indigenous]

The participant directly attributed the care she received to WelTel, highlighting the potential for WelTel to improve patient care and health outcomes:

*it’s helping me with my mental health, it’s helping me with other problems that I have and it’s all because that one little text, how are you doing.*

[Woman, Indigenous]

It should be noted that this was not the experience of all participants, as 1 participant explained:

*they try to help fix my problem or whatever by putting me in touch with somebody else, just that person can only do so much. No one can believe a magic wand.*

[Man, Indigenous]

Ultimately, while WelTel may improve clients’ connection to primary care, limitations in the health care system are still present. Furthermore, although generally perceived as beneficial, a minority of participants felt that WelTel did not impact their communication with the clinic, and some participants expressed a preference for in-person communication with clinic staff:

*I’m not a big fan of sitting or text messaging or talking to somebody on the phone. I’d rather face-to-face.*

[Man, Indigenous]

In recognizing the benefits of communicating through text message, it should be noted that texting was not all participants’ preferred method of communication.

#### Self-Assessment

For some participants, WelTel acted as a prompt to check in with themselves and assess their current health status. This was described by a participant who said:

*I can get stuck in my head, and not really realise where I’m at. Sometimes when I’ve gotten that message, I’ve been like, how are you actually doing, are you doing okay or are you struggling with stuff right now.*

[Man, White]

One participant explained that this self-assessment motivated personal action to support their overall well-being when they shared:

*the phone call says hey, how are you. Hey, I need a shower.*

[Woman, Indigenous]

A similar viewpoint was shared by another participant who felt their increased awareness of their health and wellness was inextricably linked with their primary care:

*I think this reminds me about my health and wellness because of where it came from. So, it has automatically got a subliminal message, I guess, it just makes me more aware. That’s about all because I don’t really think about Hope to Health with the regular telephone, so it’s easier to slip my mind or forget about.*

[Man, Indigenous]

While the “how are you” message does not explicitly mention the Hope to Health Clinic, receiving the automated message created awareness of the participant’s well-being and acted as a reminder to engage with the clinic.

#### Program Improvement

The WelTel program had a high degree of acceptability among participants in the study. The automated check-in messages were sent weekly on Mondays; all participants reported satisfaction with the frequency of text messages, and multiple participants stated that Mondays were an ideal day to receive the text messages. As reported by the participants, at times, the conversations that took place through WelTel contained sensitive medical information. However, all participants in the study had no privacy concerns about the WelTel platform and discussing medical issues on their mobile phones. While some participants indicated that they were receiving appointment reminders through WelTel, others indicated that they would like to see the program expanded to include appointment reminders.

There were several recommendations made regarding the messages that were exchanged between participants and the clinic staff after the initial automated check-in message. The medical office assistants at the Hope to Health Clinic monitored the incoming text messages and often engaged in text conversation with the clients. Clients would not know which clinic staff were replying to their messages, and 1 participant suggested it would be helpful if the clinic staff included their name. When clients indicated they were okay in response to the automated check-in, some reported that they did not receive any further messages, while others stated they would receive a reply.

Some participants felt follow-up messages were unnecessary when they had reported that they were okay, with 1 client stating:

*it really doesn’t make a difference. The only time it would maybe make a difference would be if I’m not okay.*

[Man, Indigenous]

Although overall well received, there did not appear to be a high demand for clinic staff to reply to text messages from clients when they indicated that they were okay.

As shown in the quantitative surveys, 59% of Weltel check-in messages received no response, and reasons given for nonresponse in the qualitative interviews included busy schedules, being preoccupied, or sleeping when messages were received. When participants did not respond to the weekly check-in messages, they did not receive communication until the following Monday. For 1 participant, this exposed a gap in the service provided:

*There have been a couple times where it has gone over a week without my responding, and no one has questioned it or anything. And I was using it as my safety net, I guess, that I’m 50 so anything could happen, I could slip in a tub, who knows whatever. So, after a few days I would have expected, hey just checking in again.*

[Man, Indigenous]

While participants were instructed at enrollment that the WelTel program was not an emergency service, providing follow-up messages to nonresponse may provide extra support while remaining within the scope of the program. Furthermore, this participant’s experience highlights how creating clear expectations at enrollment can aid clients in understanding how WelTel may be able to support their health.

Similarly, at enrollment, participants were informed that messages would receive a response within 2 business days, excluding weekends and holidays. When asked how long it takes for the clinic to respond to their messages, 1 participant reported that:

*the longest it has taken would be the next day, and the fastest would have probably been within probably a few hours. I may have gotten one or two that were half an hour or about.*

[Man, White]

This was perceived as a lag in response time by the participant when he described his experience with the WelTel check-in message:

*There’s not a lot attached to it, like, how are you. You’re either fine, not okay, or you’re doing pretty good. I found there’s quite a lag in the response time, so if I’m really not doing okay, it would be nice to hear back from someone sooner. And there’s not always a lot attached to it, if you ... I think a couple of times I’ve been like, no, I’m not having a very good day. Someone has asked, is there anything we can do to help, but other times it hasn’t happened, it’s just like, I’m sorry to hear that, hope things get better.*

[Man, White]

In addition to expressing dissatisfaction with the response time, this participant did not find the type of responses they received was consistently adequate. Recognizing limitations introduced by the workload of clinic staff, reducing the time to response, and providing additional training to clinic staff could address the concerns presented by this participant.

## Discussion

### Principal Findings

Our study provides evidence that the WelTel intervention is acceptable and has the potential to facilitate engagement in primary care for a cohort of clients facing systemic barriers in an inner-city urban low-income neighborhood where substance use, mental health disorders, and homelessness are highly prevalent. Participants were largely positive about the program, and the majority of participants had favorable views about receiving the weekly text messages. As found among a cohort of people living with HIV in Vancouver [32], participants expressed that the WelTel intervention made them feel cared for, which stands in contrast to findings that residents of the DTES face discrimination and dehumanization by primary care providers [26]. In the context of the DTES where clients face stigma and judgment, the finding that the WelTel program provided a nonjudgmental space to share health care concerns with open communication meets a basic need that had been lacking [26]. Participants were not only able to express their concerns more freely with their providers but were also made more aware of their health needs, which in turn acted as a pathway to receive care via strengthened relationship with the clinic. Additionally, the self-assessment prompted by this increased awareness may aid in detecting health issues earlier and before the progression of the health issue [15].

At times, participants’ expectations did not match the scope and/or capacity of the program, providing guidance on both how the program can be strengthened and ways in which instruction and support at the time of registration for WelTel can be improved. As opposed to 1-way mHealth texting programs, or programs that use chatbots, a benefit of WelTel is the 2-way communication approach that can facilitate discussion between clients and providers [33]; this potential for conversation was used and understood differently by clients in this study. In a similar study using WelTel, clients receive a follow-up call from a case manager if they did not respond to their check-in message by the end of the week [21], which may address nonresponse as well as align more closely with the expectation that WelTel could be used as a safety net. Text-based appointment reminders have been shown to reduce missed medical appointments [34,35], and many participants showed interest in receiving text-based appointment reminders, but only some expressed that they were currently receiving appointment reminders through WelTel. Participants reported varying experiences regarding the type of follow-up messages they received when they replied to the automated WelTel check-in, including whether they received any messages when they reported they were okay. Improving consistency regarding the type of messages clients can expect may help manage expectations and maintain quality. Additional training and workflow management could support health care providers in integrating WelTel into their daily tasks, as well as ensuring all key messages about the functionality of WelTel are communicated to clients at the time of registration.

Phone access and retention were a challenge throughout this study, mirroring similar work in the DTES that identified lost/damaged/stolen phones as the primary barrier to using WelTel [32]. A significant proportion (45%) of our participants did not have access to a phone at the HERE study baseline survey, and those who had used opioids in the past 3 months were less likely to have retained the study-supplied device or have access to a phone after 6 months. Phone retention was dynamic, and the number of functional phones participants had fluctuated throughout the study period, with some participants replacing the study-supplied phone after it was lost or stolen. The qualitative interviews primarily explored participants’ experiences with WelTel and were limited to participants who had a functional phone after the 6-month follow-up period. While WelTel was shown in this study to provide benefit for those able to use the platform, a major gap exists for those who may be facing additional vulnerabilities that create challenges for phone retention. The scalability of this pilot project may be limited by clients’ access to a mobile device, as providing a phone did not sufficiently address barriers to phone access. Notably, PRAs made considerable effort to connect with study participants for follow-up interviews but had limited success (n=33 at the 2-month follow-up), highlighting limitations in the potential for outreach using mobile phones.

This intervention aims to increase engagement for people who experience barriers to remaining attached to primary care; however, these same barriers may have impeded our teams’ ability to connect with participants selected for the pilot project. Therefore, those with the highest need may not have been included in this cohort. Participants may have felt motivated to provide positive feedback about the program because they had received a phone and talk/text/data plan. However, many participants felt comfortable sharing that they were not happy with the quality of the phones, which indicates that responder bias was mitigated in this study, likely through the inclusion of skilled PRAs who shared lived/living experience with participants. This relatively small cohort provided valuable, but limited insight into the impact of an mHealth program on engagement and retention in primary care for clients with complex, chronic health needs in a Canadian context. Further research will offer all clients at the Hope to Health Clinic enrollment in WelTel to explore the scalability of providing WelTel services as part of ongoing primary care.

### Conclusions

This study showed that the WelTel intervention was generally well received by Hope to Health Clinic clients. The intervention facilitated communication and connection between clients and health care providers and fostered positive relationships for some clients. The WelTel check-in messages have the potential to act as a prompt for self-reflection and provide pathways to receive care that otherwise would not have been used. Phone retention and access were an ongoing challenge in this study. These findings support the expansion of mHealth programs to improve engagement and retention in primary care as an innovative service delivery tool in underserved populations; however, there remains a need to explore additional solutions for individuals who are not consistently reachable via a mobile phone. In the next phase of our research, we will offer WelTel enrollment to all Hope to Health Clinic clients who have access to a mobile phone, to see if, indeed, it can assist in better communication between clients and clinic staff and ultimately result in improved retention in primary care for this population.

## Abbreviations

DTES: Downtown Eastside
HERE: Hope to Health Engagement and Retention Evaluation
mHealth: mobile health
PRA: Peer Research Associate
PWUD: people who use drugs
